# A ferrofluid-based haptic guidance system for robot-assisted endovascular procedures

**DOI:** 10.1007/s11701-025-02398-y

**Published:** 2025-05-22

**Authors:** Saket Pradhan, Dennis Kundrat, Giulio Dagnino

**Affiliations:** 1 University of Twente, Enschede, The Netherlands; 2 Fraunhofer Research Institution for Individualized and Cell-Based Medical Engineering (IMTE), Lübeck, Germany

**Keywords:** Endovascular robotics, Computer-assisted surgery, Haptic feedback, Ferrofluids

## Abstract

In the context of endovascular intervention, robot assistance provides improved instrument navigation, safety, and ergonomics compared to traditional surgical approaches. However, a significant challenge with these interventions is the lack of tactile feedback for surgeons, which is crucial for precise instrument manipulation. This paper introduces a novel concept for a ferrofluid-based haptic feedback system designed to potentially address this gap in robot-assisted endovascular surgeries. Leveraging the unique properties of ferrofluids, which alter their viscosity under magnetic fields, this system aims to mimic the tactile sensations that are otherwise lost in robotic surgeries. The study presents the development and validation of a ferrofluid-based system integrated within the CathBot, a robotic platform for endovascular procedures. The system utilizes ferrofluids in a teleoperated setup to provide real-time, intuitive feedback about instrument positioning, potentially enhancing surgical accuracy and safety. Experiments were conducted to evaluate the properties of the ferrofluids and their interaction with magnetic fields to create a responsive feedback mechanism. Results from structural experiments, force evaluations, and user studies indicate that ferrofluids have the potential to effectively providing tactile feedback through controlled magnetic fields, improving the surgeon’s ability to detect and respond to contact points within the vasculature. Despite some challenges with fluid control and system integration, the preliminary outcomes are promising. The potential improvements include refining the feedback mechanism to better mimic natural tactile sensations and further integrating this technology with existing robotic systems to enhance operational efficiency and patient safety.

## Introduction

Cardiovascular diseases (CVD) represent a significant global health challenge, manifesting through various conditions that affect the heart or blood vessels, such as ischemic heart disease and congestive heart failure. These ailments, characterized by the narrowing of blood vessels or progressive heart illness, are leading causes of mortality worldwide [1]. The primary treatment modalities for such conditions include pharmacological interventions and endovascular procedures like angioplasty, which are categorized under minimally invasive surgeries (MIS) [2]. MIS offers numerous advantages over traditional surgeries, including reduced pain, scarring, and recovery times, making them a preferable option for both patients and surgeons [3]. 

With the advent of medical robotics in the mid-1980s, robot-assisted surgeries have garnered significant interest, primarily due to their ability to enhance surgical precision beyond conventional methods and to augment the surgeon’s dexterity [4–6]. Such surgeries, often termed minimally invasive robotic surgeries (MIRS), promise shorter patient recovery times and hospital stays by minimizing interventional trauma. However, MIRS introduces new challenges, notably the disruption of surgeons’ direct hand–eye coordination and the absence of natural tactile feedback. This limitation is significant in endovascular surgeries where understanding the position and movement of instruments as well as tissue-instrument interactions within the patient’s vasculature is crucial for successful outcomes [7].

This study introduces a novel approach to overcoming the tactile feedback limitation in robot-assisted endovascular intervention by proposing a ferrofluid-based feedback system. This system is designed to integrate with the teleoperated architecture of robotic platforms, such as the CathBot [8, 9], to provide surgeons with an intuitive and tactile understanding of intravascular instrument positioning during procedures. Ferrofluids, known for their unique ability to change viscosity in response to magnetic fields, offer a promising avenue for simulating the tactile sensations lost in MIRS [10]. This concept aims to restore the natural feedback mechanisms essential for precise surgical manipulation, potentially transforming the efficacy and safety of robotic-assisted endovascular procedures [11].

This study hypothesizes that a ferrofluid-based haptic feedback system could replicate the natural tactile feedback experienced in conventional endovascular surgeries, thereby enhancing the surgeon’s ability to perform delicate endovascular procedures with higher precision and safety.

Unlike conventional motorized haptic feedback systems—which rely on electromechanical actuators that are often bulky and incompatible with MR environments [12]—ferrofluids offer a passive, MR-safe means of translating magnetic stimuli into localized deformation. Their ability to self-align along magnetic field lines allows for directional control without the need for mechanical linkages or sensors within the feedback interface. This work introduces a novel application of ferrofluid-based actuation in a compact format optimized for rendering of discrete haptic cues during catheter navigation, offering a pathway toward an MR-compatible, user-centered tactile feedback and guidance in endovascular robotic platforms. Hence, this work demonstrates the first feasibility study towards alternative feedback mechanisms mimicking established clinical interaction concepts.

The next section provides the background of the study, followed by Sect. “Methodology” presenting the methodology used to design the feedback system. Sect. “Experimental setup” provides a detailed analysis of the experimental validation, followed by results and discussion of the findings. The paper concludes by presenting potential directions for future research in this innovative field.

## Background

The advent of telerobotics and smart fluids, including ferrofluids, heralds a significant leap forward in the realm of medical feedback systems. This section delves into the foundational concepts underpinning this study, laying the groundwork for understanding the innovative approach proposed herein.

### Teleoperated surgical robots

Teleoperated surgical robots represent a pivotal advancement in minimally invasive surgery, enabling surgeons to execute complex procedures with unprecedented precision and control [4, 5]. These systems typically comprise a surgeon’s console, robotic end effectors, and a vision system, facilitating operations through small incisions. This technology offers significant advantages, including enhanced precision, reduced patient recovery times, and improved surgical ergonomics. However, challenges such as high costs, a steep learning curve, and limited tactile feedback pose barriers to widespread adoption [13].

Despite existing limitations, ongoing technological advancements and the integration of innovative features are poised to enhance the capabilities of teleoperated surgical robots further [5].

### CathBot: a robotic system for endovascular intervention

**Fig. 1:**
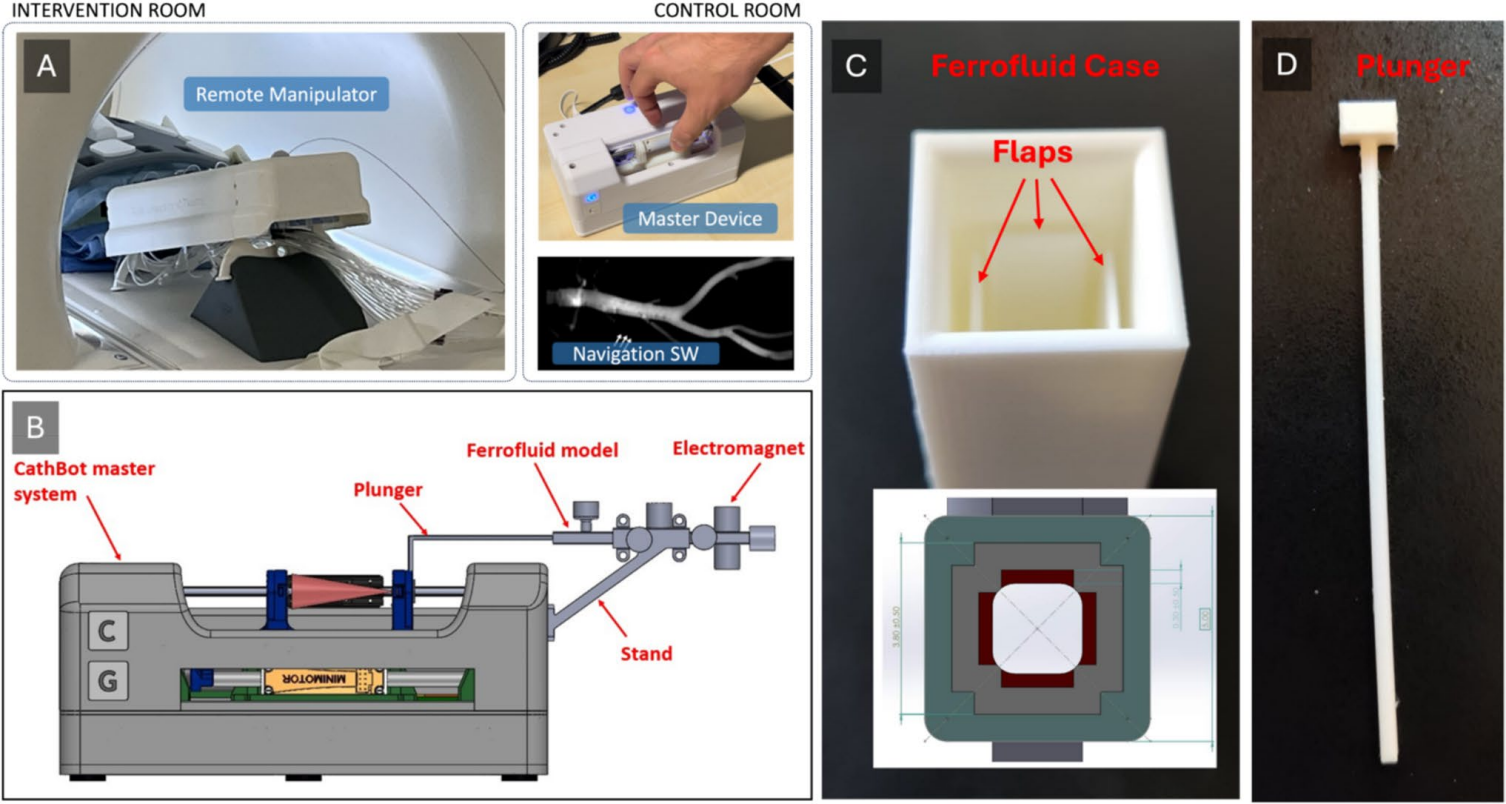
Components of the proposed ferrofluid-based haptic guidance system for robot-assisted endovascular procedures. **A** The CathBot robotic platform, composed by a master device, remote manipulator and navigation system. **B** Cross-sectional schematic of the CathBot master device with the ferrofluid-based system connected to it. **C** Close-up and schematic of the ferrofluid case featuring dynamic flaps designed to bend in response to magnetic actuation of the ferrofluid and, thus, render the structural change to the user. **D** Detailed view of the plunger component responsible for rendering the haptic feedback to the user

The CathBot platform (Fig. 1A), as introduced by Kundrat et al*.* [7–9, 14], exemplifies the application of teleoperation in medical interventions, specifically in endovascular procedures. This system comprises a primary unit controlled by the clinician and a secondary unit that interacts with the patient. This setup not only facilitates remote operations but also aims to reduce the surgeon’s exposure to radiation. Despite features for realization of haptic feedback integrated into CathBot [7], reliant on linear and rotary motors, this approach offers a glimpse into the current limitations of providing comprehensive tactile feedback in such a robotic system. Please refer to [7–9, 14] for a detailed description of the robotic system and its technical implementation.

### Haptic guidance in robotic surgery

Haptic guidance in robotic surgery is at the forefront of innovations aimed at integrating tactile feedback into teleoperated surgical systems, addressing a critical limitation: the absence of tactile sensation [15]. This technology enables surgeons to feel the texture, resistance, and force exerted by and against surgical instruments, mirroring direct tissue manipulation. Despite its potential to enhance surgical training, precision and safety, haptic guidance faces notable challenges.

In manual catheterization, clinicians rely heavily on tactile cues to detect resistance, identify anatomical landmarks, and avoid excessive force that may result in complications such as vessel wall perforation, dissection, or guidewire misplacement [16]. In robotic systems, especially those lacking real-time force feedback, these risks are compounded by the absence of haptic perception and limited visual feedback due to 2D fluoroscopy or MRI-based navigation [17].

This limitation is particularly critical in robotic cardiac interventions, where the margin for error is extremely small due to the fragility of coronary vessels and the dynamic cardiac environment. Introducing directional haptic cues—such as feedback indicating contact with the vessel wall—could help prevent over-insertion, support precise tool navigation, and reduce procedure time and complications [15].

Therefore, the development of compact haptic systems that restore this perceptual channel is not only a technical challenge but also a clinical necessity in advancing the safety and usability of robotic endovascular systems.

Current advancements include sophisticated haptic feedback mechanisms that simulate tactile sensations, advanced sensors on robotic instruments for capturing interaction data with tissues, and complex software algorithms for converting sensor data into intuitive haptic signals. These elements work together to provide surgeons with a more immersive and informative surgical experience [18–20].

However, the development and integration of these systems introduces complexity and significant cost, impacting accessibility and adoption [21, 22]. Achieving realism and accuracy in haptic feedback is challenging due to the difficulty of accurately replicating the feel of human tissues. In fact, the vast majority of commercial surgical robots do not provide haptic guidance so far due to control complexity and limited end-user acceptance.

Despite these limitations, ongoing research is making strides in overcoming these barriers, with substantial investments in improving sensor technology, feedback mechanisms, and data processing algorithms [23]. The continued evolution in this field promises to make haptic feedback a standard in future generations of surgical robots (such as the newly announced Da Vinci 5 from Intuitive Surgical, which will integrate haptic guidance), potentially transforming robotic surgery by closely replicating the traditional tactile experience of surgery, thereby enhancing outcomes through improved precision and safety as well as clinical acceptance [24].

### Clinical requirements

The need for haptics has been discussed in the endo- and neurovascular domain for over a decade. Latest clinical developments to more complex interventions have outlined the importance of integrating this technology into novel robotic platforms [16]. To meet the clinical demands of endovascular interventions, haptic systems must satisfy several key performance criteria that are determined by established interventional setups in the clinics. These include a force resolution threshold of ~ 0.05–0.1 N to match the sensitivity of the human fingertip [25], a response latency of under 100 ms for intuitive and transparent operation [26], and directional accuracy sufficient to distinguish at least four different contact directions. Moreover, systems must be compact, low-profile, and ideally MR-compatible to support emerging MRI-guided navigation platforms in intended clinical use cases. These requirements guide the design of feedback solutions capable of restoring clinically relevant perceptual cues, which remain absent in most commercially available robotic systems.

### Smart fluids and their potential

The use of ferrofluids with haptic feedback mechanisms [27] in medical robotics represents a cutting-edge innovation aimed at augmenting the tactile communication between surgeons and robotic systems [28]. Haptic feedback, crucial for replicating the sense of touch, provides essential information about interactions with surgical environments or instruments. Ferrofluids, with their unique ability to change shape, stiffness, and motion under magnetic fields, offer a novel method to create dynamic, programmable interfaces that simulate tactile sensations such as pressure and texture [29].

For teleoperated surgeries, integrating ferrofluids into haptic feedback systems could significantly enhance the surgeon’s sensory awareness, enabling more precise control over robotic instruments [30]. This improvement could lead to safer, more efficient surgical procedures. Additionally, ferrofluids could be employed in wearable haptic devices, providing surgeons with real-time tactile feedback during MIS, thus improving surgical precision and reducing risks of collateral damage to surrounding tissues [31, 32].

However, incorporating ferrofluids into medical robotics presents challenges, including achieving precise control over their magnetic responsiveness and seamlessly integrating them into existing systems, such as fluidic management. Developing algorithms for accurately simulating tactile sensations remains complex. Despite these hurdles, the potential of ferrofluids to enhance haptic feedback in medical robotics is substantial.

By addressing the current limitations in MIRS feedback mechanisms, the ferrofluid-based system proposed in this work aims to bridge the gap between the precision of robotic assistance and the intuitive control inherent in manual procedures, paving the way for safer, more efficient surgical interventions.

## Methodology

This section outlines the development of a novel ferrofluid-based feedback system designed for the master manipulator of the CathBot endovascular robot (Fig. 1B) [9]. This system aims to enhance the tactile feedback provided to surgeons, mimicking the sensations of direct catheter manipulation through innovative use of ferrofluids integrated into an existing human–machine interface for endovascular surgery.

### Working principle of the ferrofluid-based haptic feedback system

**Fig. 2:**
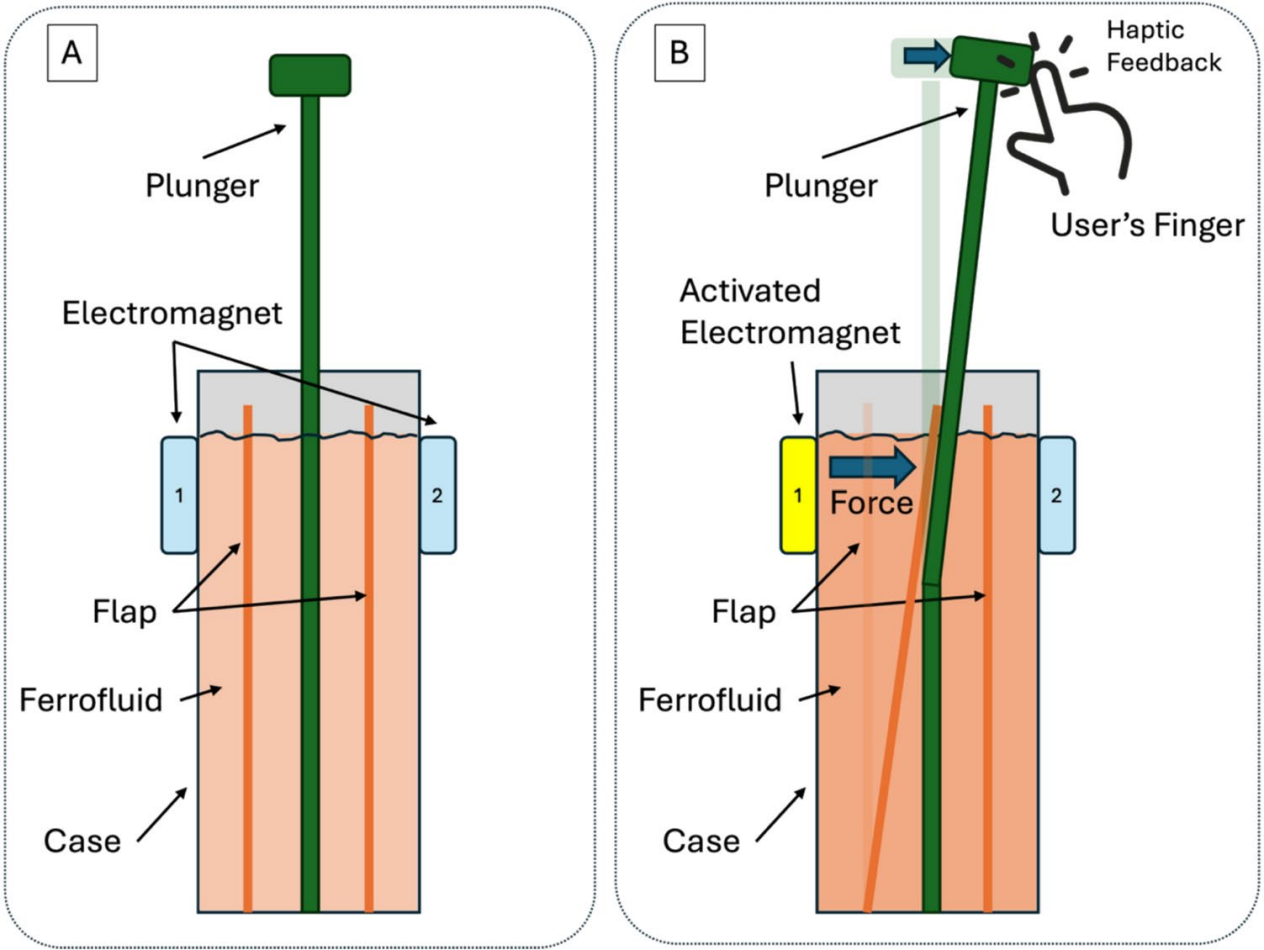
Working Principle of the Ferrofluid-Based Haptic Feedback System. **A** Schematic representation of the system, showing a cross-section view. The device includes a sealed chamber filled with ferrofluid, four internal flexible flaps, and a centrally placed plunger. Electromagnets are mounted externally on each side (only two are shown here for clarity). **B** When an electromagnet is activated (e.g., side 1), it generates a localized magnetic field that deforms the ferrofluid, increasing the local pressure. This pressure bends the adjacent flap, which elastically displaces the plunger, transmitting force to the user’s fingertips. This mechanism enables directional haptic feedback during teleoperated manipulation and, thus, a concept for the perception of directional instrument-vessel interactions

To achieve tactile feedback for CathBot users, a system that replicates the sensation of manually handling a catheter/guidewire has been developed. The fundamental concept of our haptic feedback system is to convey directional forces to the user via magnetically actuated ferrofluid. As illustrated in Fig. 2, an external electromagnet placed near the ferrofluid-filled chamber induces a deformation in the ferrofluid due to its magnetic susceptibility. This localized pressure displaces internal flexible flaps, which in turn push against a plunger element connected to the user’s hand. The plunger transmits this mechanical deflection to the fingertips, allowing the user to perceive directional feedback corresponding to the location and magnitude of catheter-wall interactions. The overall transmission pathway is as follows: electro*magnet → ferrofluid → flap → plunger → user’s finger.* This configuration aims to mimic the tactile sensation of tip contact experienced during manual catheter manipulation in endovascular procedures.

The ferrofluid used in this study was a hydrocarbon-based formulation with the following properties: saturation magnetization of 300 Gauss (30 mT), dynamic viscosity of 80 mPa·s at 27 °C, and density of 1.04 g/cm^3^ at 20 °C. The ferrofluid remained stable across a wide operating temperature range (−20 °C to + 130 °C) and had a flash point above 180 °C. These characteristics made it suitable for use in a closed chamber with magnetic actuation. The carrier fluid was a viscous black hydrocarbon oil. A volume of 0.5–1.0 mL was used per chamber, depending on test conditions.

### The ferrofluid-based haptic guidance system concept

The initial design concept involved a top-down approach, envisioning an enclosed case that simulates vasculature. It considered four primary directions—up, down, left, and right—for optimal directional feedback, leading to the conceptualization of a square case (Fig. 1C). Inside this case, flaps capable of bending 5 mm or more under forces as light as 0.0098 N were proposed. Additionally, a plunger (Fig. 1D), held by the user and connected to the CathBot’s master handle, was designed to mimic the bending movements of a catheter/guidewire within the vasculature, providing tactile feedback. The flaps’ bending, which prompts the plunger to move in specific directions, was planned to be induced by ferrofluids located between the flap and the case’s interior, as depicted in the cross-sectional view in Fig. 1C. Here, the flaps designated for directional deflection are marked in red, while the spaces set to be filled with ferrofluid are shown in gray.

Ferrofluids, influenced by an external magnetic field, generate the forces necessary for the flap’s movement. The case’s opening is sealed with a flexible, latex-based material to prevent ferrofluid leakage without adding extra force to the plungers or flaps. To eliminate air inside the case and ensure it is fully filled with ferrofluid, a vacuum chamber was designed for ferrofluid injection. Once filled, the chamber is sealed, creating a seal to prevent leakage or inward air flow.

**Fig. 3:**
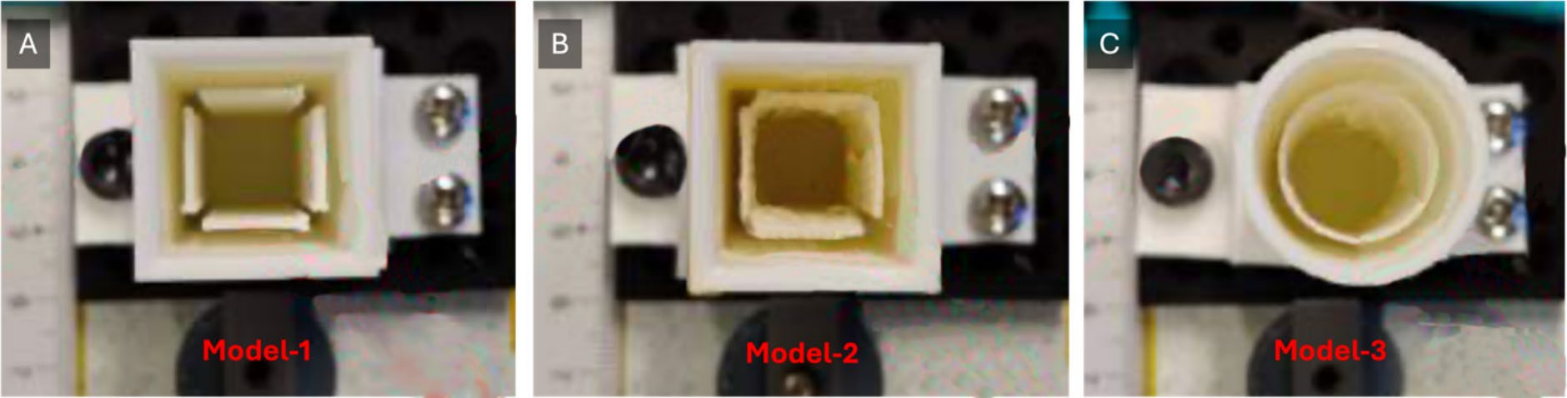
Evolution of 3D printed Ferrofluid Models for Magnetic Navigation Testing. **A** Model-1 presents a square channel within 4 flaps around the center, illustrating the initial design for magnetic manipulation studies. It is made of PLA. **B** Model-2 maintains the same geometry but incorporates flaps made of thermoplastic polyurethane presenting higher flexibility while preserving structural integrity. **C** Model-3 depicts a cylindrical chamber design made of thermoplastic polyurethane, simulating rounded vessel structures

A prototype—Model-1—was developed following the described concept design, utilizing 3D printing technology (Fig. 3A). It was crafted from white rigid Polylactic Acid (PLA), featuring an external case with a thickness of 2 mm. The flaps, measuring 0.5 mm in thickness and spaced 12 mm apart internally, were designed to replicate half the travel distance of the CathBot’s user handle at a model height of 60 mm. The flaps’ height was set at 45 mm, allowing space above them within the case for potential sealing methods.

Furthermore, two derivative models were created based on the original design. Model-2 (Fig. 3B), while maintaining the design specifications of Model-1, incorporated flaps made from thermoplastic polyurethane (TPU95A) to benefit from its enhanced flexibility. Model-3 (Fig. 3C) adopted a cylindrical shape to better resemble the anatomy of an artery, featuring an inner cylindrical flap also made from TPU95A, with the external casing produced from the same white tough PLA. The adaptations and their implications are further detailed in Sect. “Experimental setup”.

The design criteria for the plunger stipulated that it should be capable of bending under forces less than 0.0098 N while still retaining enough structural integrity to resist bending from its own weight or the pull of gravity. The dimensions of the plunger were set to a length of 100 mm and a uniform width and height of 2.5 mm each (see Fig. 1D). For usability purposes, an additional feature was incorporated at the head of the plunger to enable its attachment to an aluminum assembly, a detail further elaborated in Sect. “Experimental setup”. The plunger was fabricated using an Ultimaker S5 3D printer, utilizing TPU95A material—a filament known for yielding flexible, highly elastic 3D prints, making it suitable for items requiring rubber-like properties.

While the current prototype uses manually positioned permanent magnets to demonstrate basic functionality, the intended integration involves embedding four electromagnets around the ferrofluid chamber, each corresponding to a directional contact cue (up, down, left, right). The Ferrofluid-Based Haptic Feedback System is embedded into the CathBot manipulator (see Fig. 1B) to provide directional feedback to the operator. Control of embedded electromagnets is facilitated through hardware-specific modules connecting to the CathBot’s existing software architecture. Contact detection data from catheter image-based vessel proximity estimates [7] can be mapped to directional cues, triggering and controlling the corresponding electromagnet to induce localized ferrofluid deformation and directional haptic feedback (Fig. 2B).

## Experimental setup

**Fig. 4:**
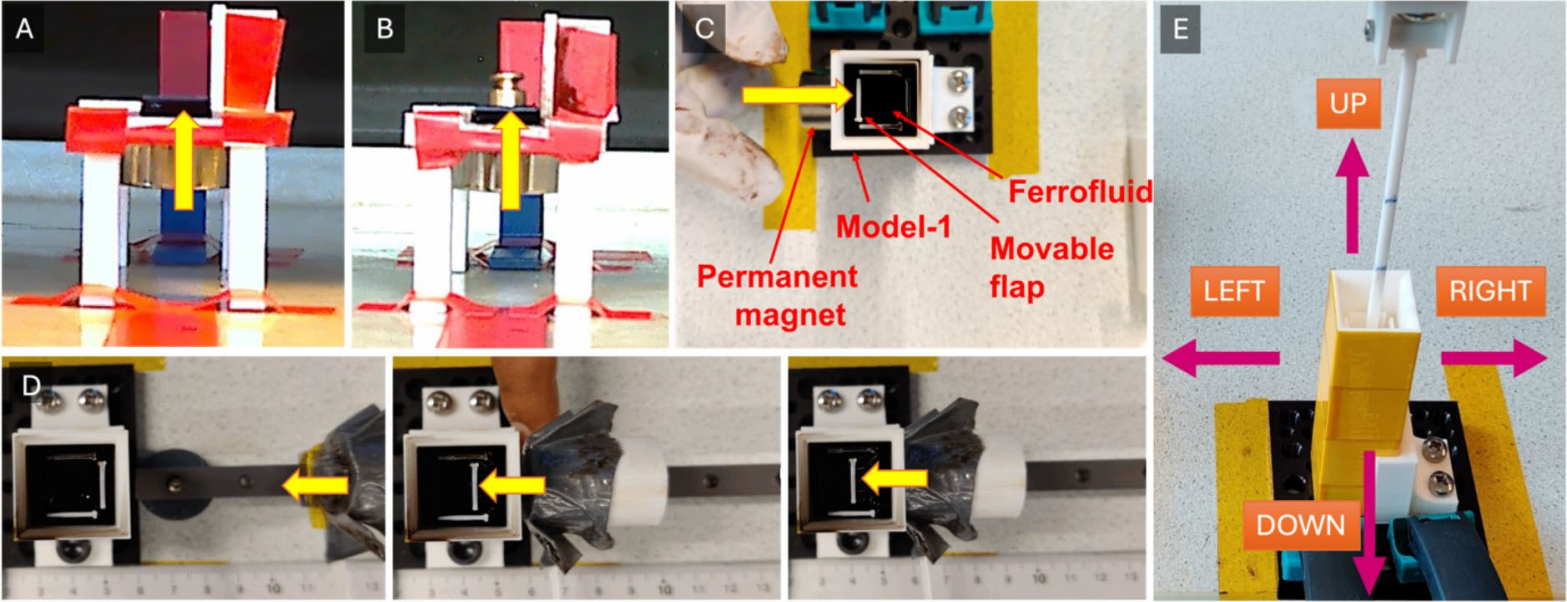
Experimental validation of the ferrofluid-based haptic guidance system. **A**, **B** Side views of the flap elevation test setup using permanent magnets to assess ferrofluid response: **A** without additional load, and **B** with a calibrated weight applied to the flap. **C** Top-down view of Model-1 showing key components, including the flexible flap and the position of the permanent magnet. **D** Top view of flap deflection under magnetic actuation, illustrating how the magnet influences ferrofluid displacement and resulting flap motion. **E** User study setup for tactile feedback evaluation, with annotated directions corresponding to the plunger’s potential deflection based on magnetically actuated ferrofluid forces within Model-1

The experimental phase of this study was structured to validate the design concept and assumptions behind the development of a ferrofluid-based feedback system for the CathBot system. This involved a series of experiments focusing on flap elevation under magnetic influence, force evaluation of the ferrofluid, and an in-depth user study to assess the system’s tactile feedback mechanism. Figure 4 summarizes the experimental setup.

### Flap elevation test

The primary objective was to confirm if ferrofluid could elevate a flap when exposed to an external magnetic field. Utilizing a 90 × 10x0.5 mm flap made of Polylactic Acid (PLA) as depicted in Fig. 4A, the test was first conducted with an electromagnet and subsequently with permanent magnets to compare the effects of varying magnetic field strengths.

**With electromagnet**—An electromagnet setup (INTERTEC ITS-MSM-1515-12VDC, 1.4W electromagnet with a maximum holding force of approximately 2.04 kg), complemented by a platform and flaps designed for flexibility and sensitivity to minimal forces, was employed to observe the ferrofluid’s behavior under magnetic influence. The arrangement allowed precise placement and observation of the flap’s elevation, which was documented visually using a LAMAX X7.1 NAOS action camera. The procedure involved introducing 0.5–1 mL of ferrofluid onto the platform and activating the electromagnet at increasing current intensities to observe the flap’s movement.

**With permanent magnet**—To explore the impact of stronger magnetic fields, the experiment was replicated with two types of permanent magnets. Modifications were made to the initial platform to prevent ferrofluid leakage, ensuring a controlled environment for observing the flap’s elevation.

Magnetic field strength values were estimated using manufacturer specifications for both the electromagnet and the permanent magnets. At approximately 1 mm from the magnet surface, the electromagnet was estimated to generate a magnetic field of ~ 80 mT at nominal current, while the disc and ring permanent magnets were estimated to generate ~ 390 mT and ~ 270 mT, respectively. These significant differences in field strengths explain the minimal flap deflection observed when using the electromagnet compared to permanent magnets, despite similar ferrofluid volumes.

### Evaluation of force exerted by ferrofluid

This experiment aimed to quantify the force exerted by ferrofluid on the flap using a weight-based setup (Fig. 4B). By incrementally adding weight to the flap and observing its elevation in response to magnetic activation, we could infer the ferrofluid’s force transmission capabilities. This setup provided valuable data on the flap’s behavior under varied weights and magnetic field strengths.

### Model testing

Model testing was conducted to determine the optimum positioning for magnets around the ferrofluid-contained models, thereby achieving the desired flap deflection. Model-1 (see Fig. 3A), underwent several iterations to fine-tune the magnetic field’s impact on ferrofluid behavior within the model.

**Experiment 1: optimum magnet position.** This experiment sought to identify the most effective placement of magnets on the model’s surface to maximize flap deflection. A series of Neodymium N42 disc magnets was strategically placed at varying heights along the model’s exterior, with flap deflections captured and analyzed to ascertain the optimum magnet positioning.

**Experiment 2: optimum magnet distance.** Focusing on the distance between the magnet and the model, this experiment evaluated how the proximity of different magnets influenced flap deflection. Models were designed with varying compositions and shapes to assess the ferrofluid’s responsiveness across a spectrum of magnetic field intensities and distances (Fig. 4D).

### User study

In a user study with 10 participants, including 9 university students and a researcher (non-clinical), we evaluated the feasibility of tactile recognition of plunger deflection caused by magnetic field-induced flap movement in the developed device (Fig. 4E). The aim was to test whether participants could discern its bending and direction by touch.

Participants engaged in four test variations involving different combinations of finger positioning (index-thumb, ring-little finger) and eye state (open, closed). Namely:

1. Index and Thumb, Blindfolded (ITB)
2. Index and Thumb (IT)
3. Ring and Little finger, Blindfolded (RLB)
4. Ring and Little finger (RL)

Each variation was repeated 10 times, with the setup including an aluminum rod framework and the device filled with 10 ml of ferrofluid, secured to restrict movement.

Instructions emphasized a relaxed grip, central alignment of the plunger, and avoidance of looking at the setup during tests. A permanent magnet S-20–20-N provided the magnetic field. Test order and magnet direction were randomized. Post-experiment, participants completed a questionnaire, with results discussed in the subsequent section.

## Results

### Flap elevation test

**With electromagnet**—The initial observations from the flap elevation test utilizing an electromagnet indicated that the presence of ferrofluid already elevated the flap even before electromagnet activation, with no significant visual elevation upon electromagnet activation. The analysis revealed a minimal elevation change of 0.01 mm (2.5% higher at maximum power), which is significantly lower than the ideal range (0.5–2.67 mm) necessary for effective tactile feedback.

**Fig. 5:**
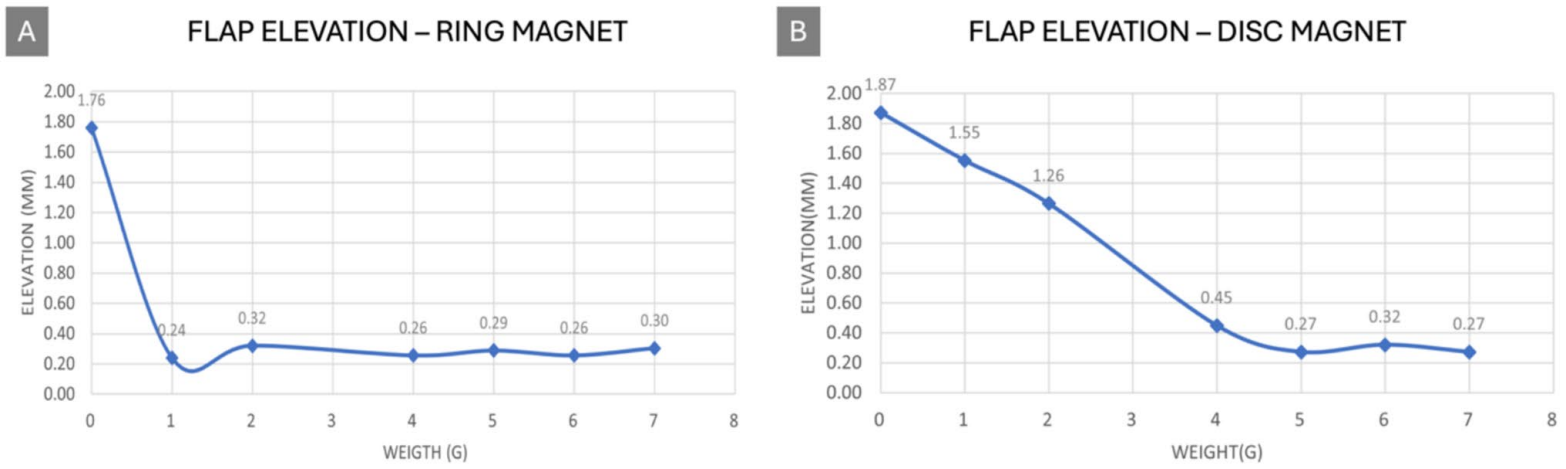
Flap elevation response to magnetic actuation with varied weights. **A** The elevation of the flap in response to a ring magnet across different weights demonstrating a sharp decline in elevation as weight is applied. **B** The response of the flap elevation to a disc magnet, exhibiting a more gradual decline and a stabilization of elevation with increasing weights, showing slightly higher elevation values than with the ring magnet. These graphs illustrate the flap’s magnetic responsiveness and the effect of magnet type on the flap’s deflection under incremental loading

**With permanent magnet**—Switching to permanent magnets with higher magnetic field strengths, the observed flap elevation was more pronounced. With a ferrofluid volume of 1 ml, no elevation change was recorded with both ring and a disc magnet. With a ferrofluid volume of 2 ml, and an elevation of up to 1.77 mm and 1.89 mm were achieved for a ring magnet and a disc magnet, respectively, underscoring the importance of magnetic field strength and fluid volume in achieving desired elevation levels (Fig. 5).

### Evaluation of force exerted by ferrofluid

The evaluation highlighted that the ferrofluid could exert a measurable force on the flap (Fig. 5), with the force increasing significantly with stronger magnets. The observed flap deflections were interpreted using a cantilever beam approximation to estimate the force required to produce the measured displacements. Assuming a uniform PLA beam with fixed support and known dimensions (length 90 mm, thickness 0.5 mm), the force was estimated using the Euler–Bernoulli beam equation. For the maximum observed deflection of ~ 1.89 mm with the disc magnet (S-20-08-N), the calculated tip force was approximately 0.061 N – notably higher than that for the ring magnet (CS-S-18-04-N), which only managed 0.0122 N, indicating the magnetic field strength’s critical role in the force exerted by the ferrofluid. This value exceeds the conservative human tactile threshold (~ 0.05 N), indicating that the flap’s motion is likely sufficient to generate a perceivable sensation through the plunger [25].

### Model testing

Model testing confirmed that flap deflection increased as the magnet moved closer to the model’s opening, with stronger magnets inducing greater deflection. This finding suggests that magnet positioning and strength are crucial for maximizing flap deflection and, by extension, tactile feedback.

**Fig. 6:**
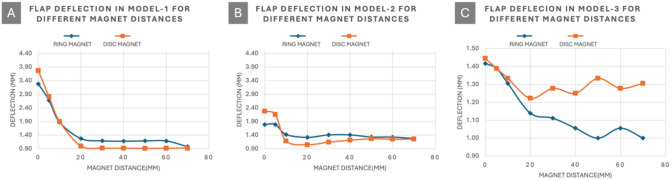
Comparative analysis of flap deflection across three ferrofluid-based models at varying magnet distances. **A** Model-1 demonstrates a steep initial deflection that diminishes rapidly as the distance between the magnet and the chamber increases. **B** Model-2 shows a similar deflection trend, though with lower overall displacement, attributed to the increased flexibility of the thermoplastic polyurethane (TPU95A) flaps. **C** Model-3 exhibits a less consistent deflection pattern, likely due to the cylindrical chamber geometry introducing non-uniform magnetic field interactions. These results highlight the critical role of magnet positioning and geometry in optimizing ferrofluid-based actuation for haptic feedback systems in robotic surgical applications

Deflection was negligible until the magnet approached within 20 mm of the model surface, indicating a threshold distance for effective magnetic interaction with the ferrofluid. Notably, flexible flaps (Model-2) exhibited non-uniform deflection, complicating the determination of optimal magnet distance (Fig. 6).

The observed flap deflection saturation beyond ~ 20 mm magnet distance (Fig. 6) aligns with the known nonlinear decay of magnetic field strength from permanent magnets. While no FEM simulation was conducted in this study, the experimental behavior matches the inverse-cube decay trend typically associated with axial field lines from cylindrical magnets [33]. This suggests that beyond a critical air gap (~ 20 mm), the magnetic pressure exerted on the ferrofluid falls below the threshold required for effective flap deflection. In future work, we plan to conduct FEM analysis to optimize magnet geometry and placement, enabling more uniform and predictable actuation performance across a wider range of operating distances.

### User study

The study aimed to determine if participants could detect movement and its direction in the plunger due to interactions with Model-1. Results were summarized in two tables: Table 1 outlines volunteers’ ability to identify the bending direction, while Table 2 details compliance across different experimental conditions described in Sect. “Experimental setup”. Compliance was defined as correctly sensing both the movement and its intended direction. Incorrect sensing occurred when the movement was detected, but the perceived direction differed from the intended one. A “NO” response indicated no movement was detected, with Table 1 showing that direction “UP” (frontward) had the highest compliance at 60%, and “LEFT” the lowest at 29%.

**Table 1:** User study results: sensed directions and corresponding direction-based compliance

		Sensed direction					Compliance	
		Up	Down	Right	Left	No	Sensed	Not sensed
Actual direction	Up	**70**	2	18	5	21	60%	18%
	Down	1	**30**	8	8	43	33%	48%
	Right	9	16	**53**	0	33	48%	30%
	Left	18	2	7	**24**	32	29%	39%

Bold values identify the correct direction detection (i.e., full compliance) for each movement condition

**Table 2:** User study results: compliance for different variations

Variation	Number of instances	Compliance
ITB	100	64%
IT	100	62%
RLB	100	34%
RL	100	28%

Overall, volunteers detected plunger movement 68% of the time, with accurate direction identification 44% of the time. “DOWN” was the most frequently undetected direction. Error patterns showed a rightward bias in incorrect direction sensing, implying a clockwise error tendency around the intended direction. Table 2 revealed that the variation ITB had the highest compliance at 64%, whereas RL had the lowest. Index finger and thumb variations generally showed higher compliance than those involving the ring and little fingers.

Overall, the results from the flap elevation tests, force evaluation, model testing, and user study collectively validate the feasibility of employing ferrofluid and magnetic fields to achieve tactile feedback in robotic surgery systems. The findings highlight the importance of optimizing ferrofluid volume, magnet positioning, and magnetic field strength to enhance the system’s effectiveness and user experience.

## Discussion

This work explored a novel application of ferrofluid in medical feedback systems, showcasing through validation experiments that ferrofluid can indeed cause flap elevation by aligning with the magnetic field lines of a permanent magnet. Although a similar electromagnet could potentially achieve the same effect, its weaker strength in the tests limited the observed flap elevation. Notably, flaps with oleophobic coating unexpectedly attracted more ferrofluid stains than those without coating.

Significant insights were gained from using permanent magnets, particularly the observation of a 400% increase in flap elevation when the ferrofluid volume was doubled, as ferrofluid arranged itself along the magnetic field lines, replicating the magnet’s shape to some extent. This finding underscores ferrofluid’s potential in actuation-based applications, though the force it exerts was estimated due to simplifying assumptions such as point load forces and uniform bending. These preliminary estimations, while simplified, provide insight into the potential of the system to deliver forces within perceptible ranges. However, they do not account for material damping, friction, or non-uniform flap deformation. Future iterations will include embedded force sensors and dynamic system modeling to validate and refine force predictions under realistic operational conditions. Extended work will also include novel control concepts based on integrated sensing, non-linear material and structural modeling to improve force rendering to the operator.

Following the experiment’s success, Model-1 was created with thin white tough PLA flaps to examine deflection at various magnet positions. The study found that positions closer to the model’s face resulted in higher deflection up to a distance of 45 mm. Model-2 introduced TPU95A flaps for greater flexibility and deflection, though results varied due to the material’s properties. A cylindrical design in Model-3, aiming to mimic an artery’s cross-section, showed resistance to deformation, suggesting vertical grooves might enhance flexibility.

User studies with Model-1 highlighted the plunger’s design issues, affecting users’ ability to correctly perceive direction changes. Quick magnet introduction significantly improved direction perception accuracy compared to gradual approaches, suggesting manual magnet introduction and the researcher’s positioning could introduce biases, as noted in Table 1. Implementing electromagnets with high field strengths could offer a solution by providing instantaneous and/or dynamic magnetic fields, thereby improving accuracy. Feedback preference favored using the index finger and thumb over the ring and little finger for manipulating the plunger, likely due to the setup’s vertical orientation making the latter grip less ergonomic. Addressing these issues by redesigning the plunger and employing electromagnets could further reduce errors and enhance the system’s effectiveness. While the user study demonstrated the feasibility of directional feedback with a detection accuracy of 68%, it was conducted on a limited participant pool (n = 10) composed entirely of non-clinical users. As such, the results should be interpreted as an initial proof-of-concept rather than a clinical usability assessment. Future studies will recruit surgical trainees and interventional clinicians to evaluate performance in simulated vascular environments with more realistic anatomical constraints and stress conditions. This will help assess usability, feedback clarity, and learning curve in settings more representative of actual surgical workflows.

To contextualize the tactile feedback capabilities of our system, Table 3 summarizes key performance metrics compared to other haptic feedback technologies specifically developed for endovascular robotic interventions. The maximum force estimated from our flap deflection (~ 0.061 N) falls within the tactile detection threshold range of human fingertips (typically ~ 0.05–0.1 N), suggesting that the system can provide perceivable tactile cues under ideal conditions [25].

**Table 3:** Comparison of haptic feedback modalities in endovascular robotics

Technology	Max output force	Refresh rate	MR-compatible
This work (ferrofluid-based system)	~ 0.06 N		Yes
MR fluid-based haptic interface [35]	0.5–2 N	NA	Limited
Magnetorheological fluid-based system [34]	0.5–2 N	NA	Limited
SEA-based haptic master controller [36]	1–5 N	NA	No

Magnetorheological (MR) fluid-based systems, such as those proposed by Zhang et al*.* [34], can deliver higher continuous force outputs (up to 2 N). Similarly, haptic feedback systems using magnetically controlled MR clutches, like those in Li et al*.* [35], achieve directional guidance with moderate fidelity and complexity. However, both require more elaborate control architectures and may face limitations in MR environments.

Series Elastic Actuator (SEA)-based hand controllers, as developed by Wang and Guo [36], can generate forces up to 5 N and provide multi-DOF feedback, but at the expense of mechanical and control system complexity.

In contrast, our passive ferrofluid approach is potentially simple, inherently MR-compatible, and can be miniaturized with fewer moving parts. This positions our solution as a promising candidate for low-frequency, directional haptic events—such as wall contact cues—rather than high-fidelity continuous force rendering.

The proposed haptic feedback module is designed to interface with the CathBot’s teleoperation framework in an event-based control scheme. During navigation, catheter-tip proximity to the vessel wall can be monitored through vision-based feedback and real-time force estimation algorithms [7, 9]. When a threshold is exceeded (e.g., catheter tip < 1 mm from vessel wall), the system activates the electromagnet located on the corresponding side of the ferrofluid chamber. This induces deformation of the flap and causes the plunger to deflect in the matching direction, providing the operator with a directional warning, i.e., to initiate a countermotion for enhanced procedural safety. This approach prioritizes intuitive, low-complexity feedback rather than continuous force rendering, and is compatible with real-time constraints imposed by image-based sensing latency. Future iterations will explore integration with AI-assisted decision modules and dynamic thresholds based on context information from anatomical models.

The CathBot master device operates at a sampling rate of 100 Hz [9], which defines the available bandwidth for real-time haptic feedback integration. Although the current proof-of-concept system was tested using manually applied magnets, future implementations using electromagnets will be capable of operating within this 100 Hz control loop or even beyond. This rate is sufficient for delivering timely directional cues during catheter navigation.

In contrast, systems designed for continuous, high-fidelity haptic feedback typically require actuation and sensing rates of 300–1000 Hz to ensure transparency and simulate fine tool–tissue interactions. As our approach is intended for discrete, event-triggered feedback—such as alerts for wall contact—rather than continuous force rendering, the 100 Hz bandwidth of CathBot at lower resulting forces is considered appropriate for this use case.

Thermal stability is important to consider for clinical translation and usability of the proposed solution. Ferrofluids are known to exhibit temperature-dependent changes in viscosity, which may affect their responsiveness to magnetic fields during prolonged or high-intensity actuation. In our benchtop experiments, which employed short-duration magnet exposure and low actuation duty cycles, no significant heating or performance drift affection our experimental outcomes were observed. However, we recognize that in future iterations involving continuous or rapid electromagnet actuation, localized heating could become a concern. To mitigate this, several strategies are under consideration:

*Use of temperature-stable ferrofluids* specifically formulated for low-viscosity variation across a clinically relevant operating range (e.g., 20–37 °C);

*Thermal insulation of electromagnet cores* and optimization/adjustment of duty cycles to reduce excess heat transfer to the fluid;

*Incorporation of passive or active cooling mechanisms* (e.g., heat sinks, phase-change materials) in the chamber design or other components of the electro-mechanical setup;

*Real-time thermal monitoring and control*, using embedded temperature sensors to compensate for viscosity drift in control algorithms.

These measures will ensure long-term stability and reproducibility of haptic feedback under realistic, dynamic clinical conditions.

## Conclusion: limitations and future directions

This study presented the design, implementation, and validation of a ferrofluid-based haptic feedback system tailored for the CathBot endovascular robotic platform. The system leverages localized magnetic actuation of ferrofluids to provide directional tactile cues to the operator through a flexible plunger mechanism. Through a series of benchtop experiments—including flap deflection analysis, force estimation, and a user study—we demonstrated and pioneered the conceptual feasibility of generating perceptible haptic feedback without compromising MR compatibility or system compactness.

Although the prototype achieved a directional detection accuracy of 44% and an overall movement detection rate of 68% among non-clinical users, these results reflect early-stage development. The current configuration, which relies on manual magnet placement and vertical system orientation, limits its usability and responsiveness. Nonetheless, the findings confirm that the ferrofluid-based actuation mechanism can generate forces within the tactile perception threshold (≥ 0.05 N) and relevant clinical requirements, indicating the system’s potential as a lightweight, event-driven haptic module for robotic navigation.

To enhance performance and usability, future work will focus on four key directions:

*Plunger design optimization:* A redesigned plunger will feature a rigid proximal segment for stable grip, and a flexible distal section (e.g., TPU95A) to better transmit directional motion to the user’s fingertips. Reinforced materials or hybrid composites may be used to reduce unintentional bending from hand pressure. Besides, a simulation-driven design strategy, e.g., coupled mechanical and electromagnetic finite element analysis, will be considered to realize under given constraints an optimal design concept for physical prototyping. In particular, plunger distances and dimensions will be optimized to increase perceivable interaction forces as a function of applied magnetic field strengths.

*System reorientation and sealing:* The model will be reconfigured horizontally to improve ergonomics and align with the expected hand posture during teleoperation. This redesign requires a sealing solution that is compliant, ferrofluid-compatible, and minimally resistive to flap motion. Latex membranes and silicone-based materials (e.g., Dragon Skin) are being considered. Studies will be conducted to identify optimal sealing design with low-friction profiles.

*Electromagnetic actuation and control:* The next prototype will integrate a set of electromagnets for rapid, dynamic and controllable actuation. These will be driven by a microcontroller-based system connected to the existing CathBot framework capable of selectively triggering directional feedback based on real-time catheter-tip proximity data. This will replace the manual magnet setup and allow automated, event-based operation.

*Integration with image-based feedback:* Building on CathBot’s existing imaging and navigation framework, a control logic will be implemented to activate the corresponding electromagnet when the catheter approaches the vessel wall beyond a predefined threshold. This will ensure directional feedback is only provided when clinically relevant, enhancing precision and reducing cognitive load while maintaining clinical routines.

Finally, broader user validation—including larger trials with surgical trainees and clinical experts (n > 20) in simulated vascular environments—will be conducted to evaluate system ergonomics, learning curve, and effectiveness in supporting endovascular navigation using assistive guidance technologies. These developments will guide the transformation of this ferrofluid-based solution from a proof-of-concept to a deployable haptic interface for MR-compatible robotic surgery.

## Data Availability

No datasets were generated or analysed during the current study.

## References

[1] Vaduganathan M, Mensah GA, Turco JV, Fuster V, Roth GA (2022) The global burden of cardiovascular diseases and risk. J Am Coll Cardiol 80:2361–2371 36368511 10.1016/j.jacc.2022.11.005

[2] Dagnino G, Kundrat D (2024) Robot-assistive minimally invasive surgery: trends and future directions. Int J Intell Robot Appl. 10.1007/s41315-024-00341-2

[3] Ilcheva L, Risteski P, Tudorache I et al (2023) Beyond conventional operations: embracing the era of contemporary minimally invasive cardiac surgery. J Clin Med 12:7210 38068262 10.3390/jcm12237210 PMC10707549

[4] Troccaz J, Dagnino G, Yang G-Z (2019) Frontiers of medical robotics: from concept to systems to clinical translation. Annu Rev Biomed Eng 21:193–218 30822100 10.1146/annurev-bioeng-060418-052502

[5] Guo Y, Dagnino G, Yang G-Z (2023) Medical robotics: history, challenges, and future directions. Springer Nature, Singapore

[6] Dagnino G, Georgilas I, Tarassoli P, Atkins R, Dogramadzi S (2015) Design and real-time control of a robotic system for fracture manipulation. EMBC 2015 conference. Milan, Italy 10.1109/EMBC.2015.7319483 26737383

[7] Dagnino G, Liu J, Abdelaziz MEMK, Chi W, Riga C, Yang GZ (2018) Haptic feedback and dynamic active constraints for robot-assisted endovascular catheterization. 2018 IEEE/RSJ international conference on intelligent robots and systems (IROS). Madrid, Spain

[8] Dagnino G, Kundrat D, Kwok TMY et al (2022) In-vivo validation of a novel robotic platform for endovascular intervention. IEEE Trans Biomed Eng. 10.1109/TBME.2022.3227734 10.1109/TBME.2022.3227734 37015473

[9] Kundrat D, Dagnino G, Kwok TMY et al (2021) An MR-safe endovascular robotic platform: design, control, and ex-vivo evaluation. IEEE Trans Biomed Eng 68:3110–3121 33705306 10.1109/TBME.2021.3065146

[10] Fan X, Zhang Y, Wu Z et al (2023) Combined three dimensional locomotion and deformation of functional ferrofluidic robots. Nanoscale 15:19499–19513 37982182 10.1039/d3nr02535g

[11] Ji Y, Dai Y, Chen D, Gan C, Wang L, Feng L (2021) Precise control of ferrofluid droplet robot in 3-D vascular model. 2021 WRC symposium on advanced robotics and automation (WRC SARA), p 122–7

[12] Bijlsma J, Kundrat D, Dagnino G (2024) MR-based navigation for robot-assisted endovascular procedures. Int J Intell Robot Appl. 10.1007/s41315-024-00340-3

[13] Kim M, Zhang Y, Jin S (2023) Soft tissue surgical robot for minimally invasive surgery: a review. Biomed Eng Lett 13:561 37872994 10.1007/s13534-023-00326-3 PMC10590359

[14] Benavente Molinero M, Dagnino G, Liu J et al. (2019) Haptic guidance for robot-assisted endovascular procedures: implementation and evaluation on surgical simulator, Macau

[15] Bergholz M, Ferle M, Weber BM (2023) The benefits of haptic feedback in robot assisted surgery and their moderators: a meta-analysis. Sci Rep 13:19215 37932393 10.1038/s41598-023-46641-8 PMC10628231

[16] Gravino G (2025) Haptic feedback in robotic endovascular neurosurgical intervention: a necessity or a commodity? Interv Neuroradiol. 10.1177/15910199241304851 40101281 10.1177/15910199241304851 PMC11920981

[17] Pescio M, Kundrat D, Dagnino G (2025) Endovascular robotics: technical advances and future directions. Minim Invasive Ther Allied Technol. 10.1080/13645706.2025.2454237 39835841 10.1080/13645706.2025.2454237

[18] Mahvash M, Okamura A (2007) Friction compensation for enhancing transparency of a teleoperator with compliant transmission. IEEE Trans Robot Publ IEEE Robot Autom Soc 23:1240–1246 10.1109/TRO.2007.909825 PMC2877600 20514151

[19] Saracino A, Deguet A, Staderini F et al (2019) Haptic feedback in the da Vinci research kit (dVRK): a user study based on grasping, palpation, and incision tasks. Int J Med Robot Comput Assist Surg 15:e1999 10.1002/rcs.1999 30970387

[20] Chua Z, Okamura AM (2023) A modular 3-degrees-of-freedom force sensor for robot-assisted minimally invasive surgery research. Sensors 23:5230 37299958 10.3390/s23115230 PMC10255999

[21] Marcus HJ, Hughes-Hallett A, Payne CJ et al (2017) Trends in the diffusion of robotic surgery: a retrospective observational study. Int J Med Robot Comput Assist Surg 13:e1870 10.1002/rcs.1870 PMC5725725 29105982

[22] Marcus HJ, Payne CJ, Hughes-Hallett A et al (2016) Regulatory approval of new medical devices: cross sectional study. BMJ 353:i2587 27207165 10.1136/bmj.i2587 PMC4875244

[23] Chi W, Dagnino G, Kwok T et al (2020) Collaborative robot-assisted endovascular catheterization with generative adversarial imitation learning

[24] Bao X, Guo S, Xiao N et al (2018) Operation evaluation in-human of a novel remote-controlled vascular interventional robot. Biomed Microdevice 20:34 10.1007/s10544-018-0277-5 29627886

[25] Okamura AM, Verner LN, Reiley CE, Mahvash M (2011) Haptics for robot-assisted minimally invasive surgery. In: Kaneko M, Nakamura Y (eds) Robotics research. Springer, Berlin, Heidelberg, pp 361–372

[26] Coles TR, Meglan D, John NW (2011) The role of haptics in medical training simulators: a survey of the state of the art. IEEE Trans Haptics 4:51–66 26962955 10.1109/TOH.2010.19

[27] Kastor N, Dandu B, Bassari V, Reardon G, Visell Y (2023) Ferrofluid electromagnetic actuators for high-fidelity haptic feedback. Sens Actuators A Phys 355:114252

[28] Hooshiar A, Payami A, Dargahi J, Najarian S (2021) Magnetostriction-based force feedback for robot-assisted cardiovascular surgery using smart magnetorheological elastomers. Mech Syst Signal Process 161:107918

[29] Rørvik SB, Auflem M, Dybvik H, Steinert M (2021) Perception by palpation: development and testing of a haptic ferrogranular jamming surface. Front Robot AI 8:745234 34651019 10.3389/frobt.2021.745234 PMC8505531

[30] Shabaniverki S, Xie S, Ren J, Juárez JJ (2021) Soft ferrofluid actuator based on 3D-printed scaffold removal. 3D Print Addit Manuf 8:126–135 36655058 10.1089/3dp.2020.0012 PMC9828599

[31] Selim M, Dresscher D, Abayazid M (2024) A comprehensive review of haptic feedback in minimally invasive robotic liver surgery: advancements and challenges. Int J Med Robot Comput Assist Surg 20:e2605 10.1002/rcs.2605 38071613

[32] Yin J, Hinchet R, Shea H, Majidi C (2021) Wearable soft technologies for haptic sensing and feedback. Adv Func Mater 31:2007428

[33] Magnetic moment (2025) Wikipedia. https://en.wikipedia.org/w/index.php?title=Magnetic_moment&oldid=1277199692. Accessed 2 May 2025

[34] Zhang L, Gu S, Guo S, Tamiya T (2021) A magnetorheological fluids-based robot-assisted catheter/guidewire surgery system for endovascular catheterization. Micromachines 12:640 34070909 10.3390/mi12060640 PMC8226888

[35] Li X, Guo S, Shi P, Jin X, Kawanishi M (2022) An endovascular catheterization robotic system using collaborative operation with magnetically controlled haptic force feedback. Micromachines 13:505 35457811 10.3390/mi13040505 PMC9029488

[36] Wang K, Mai X, Xu H, Lu Q, Yan W (2020) A novel SEA-based haptic force feedback master hand controller for robotic endovascular intervention system. Int J of Med Robot Comput Assist Surg 16:e2109 10.1002/rcs.2109 32306455

